# Association between Combined Lifestyle Factors and Non-Restorative Sleep in Japan: A Cross-Sectional Study Based on a Japanese Health Database

**DOI:** 10.1371/journal.pone.0108718

**Published:** 2014-09-30

**Authors:** Minako Wakasugi, Junichiro James Kazama, Ichiei Narita, Kunitoshi Iseki, Toshiki Moriyama, Kunihiro Yamagata, Shouichi Fujimoto, Kazuhiko Tsuruya, Koichi Asahi, Tsuneo Konta, Kenjiro Kimura, Masahide Kondo, Issei Kurahashi, Yasuo Ohashi, Tsuyoshi Watanabe

**Affiliations:** 1 Center for Inter-organ Communication Research, Niigata University Graduate School of Medical and Dental Sciences, Niigata, Japan; 2 Department of Clinical Nephrology and Rheumatology, Niigata University Graduate School of Medical and Dental Sciences, Niigata, Japan; 3 Steering Committee for “Design of the comprehensive health care system for chronic kidney disease (CKD) based on the individual risk assessment by Specific Health Checkups,” Fukushima, Japan; 4 iAnalysis LLC, Tokyo, Japan; 5 Department of Integrated Science and Engineering for Sustainable Society, Chuo University, Tokyo, Japan; University of Pennsylvania Perelman School of Medicine, United States of America

## Abstract

**Background:**

Although lifestyle factors such as cigarette smoking, excessive drinking, obesity, low or no exercise, and unhealthy dietary habits have each been associated with inadequate sleep, little is known about their combined effect. The aim of this study was to quantify the overall impact of lifestyle-related factors on non-restorative sleep in the general Japanese population.

**Methods and Findings:**

A cross-sectional study of 243,767 participants (men, 39.8%) was performed using the Specific Health Check and Guidance System in Japan. A healthy lifestyle score was calculated by adding up the number of low-risk lifestyle factors for each participant. Low risk was defined as (1) not smoking, (2) body mass index<25 kg/m^2^, (3) moderate or less alcohol consumption, (4) regular exercise, and (5) better eating patterns. Logistic regression analysis was used to examine the relationship between the score and the prevalence of non-restorative sleep, which was determined from questionnaire responses. Among 97,062 men (mean age, 63.9 years) and 146,705 women (mean age, 63.7 years), 18,678 (19.2%) and 38,539 (26.3%) reported non-restorative sleep, respectively. The prevalence of non-restorative sleep decreased with age for both sexes. Compared to participants with a healthy lifestyle score of 5 (most healthy), those with a score of 0 (least healthy) had a higher prevalence of non-restorative sleep (odds ratio, 1.59 [95% confidence interval, 1.29–1.97] for men and 2.88 [1.74–4.76] for women), independently of hypertension, hypercholesterolemia, diabetes, and chronic kidney disease. The main limitation of the study was the cross-sectional design, which limited causal inferences for the identified associations.

**Conclusions:**

A combination of several unhealthy lifestyle factors was associated with non-restorative sleep among the general Japanese population. Further studies are needed to establish whether general lifestyle modification improves restorative sleep.

## Introduction

A combination of healthy lifestyle factors, such as abstaining from smoking, maintaining a body mass index (BMI) of less than 25 kg/m^2^, consuming alcohol moderately, exercising regularly, and having a healthy diet, is reportedly associated with a significantly reduced risk of developing several diseases, such as coronary heart disease [1]–[2], type 2 diabetes mellitus [3], stroke [4], sudden cardiac death [5], chronic kidney disease [6], cancer [7]–[9], and total mortality [10]. A clear linear relationship was observed in these studies between risk reduction and the number of healthy lifestyle factors, suggesting that an analysis of combined lifestyle factors may demonstrate their influence better than analyses based on single factors due to the complexity and multiple dimensions of habitual health behaviors. In addition, maintaining an overall healthy lifestyle throughout young adulthood was strongly associated with a low cardiovascular disease risk profile in middle age regardless of sex, race, or a parental history of myocardial infarction, suggesting that genetic factors may not be very important in determining a low risk profile [11]. Most of these studies were conducted in non-Japanese populations except for a few studies [6], [9]; however, a combination of healthy lifestyle factors may play a prominent role regardless of sex, race, or genetics.

Little is known about the impact of combined lifestyle factors on inadequate sleep. There is growing evidence that inadequate sleep, which includes short sleep duration and poor sleep quality, is associated with lifestyle factors that include obesity, insufficient physical exercise, and consumption of substances such as caffeine, alcohol, and nicotine [12]. Inadequate sleep may also modify eating patterns, thereby mediating or contributing to the observed relationship between sleep disturbance and obesity [13].

Inadequate sleep is associated with several chronic diseases. Epidemiological studies have shown that short sleep duration is associated with a higher risk of lifestyle-related diseases such as obesity [14]–[17], type 2 diabetes mellitus [18]–[19], hypertension [20], dyslipidemia [21], coronary heart disease [22], and chronic kidney disease [23]. Sleep quality is important in modifying the association between sleep duration and these diseases [24]–[26].

We hypothesized that a combination of unhealthy lifestyle factors is associated with inadequate sleep. Evidence of a relationship could have important clinical and public health implications. If a combination of unhealthy behaviors is associated with inadequate sleep, lifestyle interventions have the potential to reduce its occurrence. We present the results of a large cross-sectional study on the prevalence of non-restorative sleep (NRS), typically defined as subjectively feeling unrefreshed upon waking [27], and its association with a combination of lifestyle factors in the general Japanese population.

## Methods

### Study population and design

This cross-sectional study used baseline data from a prospective cohort study of 667,218 participants, aged 40 to 74 years, obtained from the Japanese Specific Health Check and Guidance System (SHC) created in 2008. Twenty-four of the prefectures participating in this nationwide project (Hokkaido, Miyagi, Yamagata, Fukushima, Ibaraki, Tochigi, Tokyo, Saitama, Kanagawa, Niigata, Nagano, Ishikawa, Gifu, Osaka, Okayama, Tokushima, Kochi, Fukuoka, Saga, Nagasaki, Oita, Kumamoto, Miyazaki, and Okinawa) agreed to participate in our study and were included in the present analysis. Data were sent to and verified by an independent data center, the NPO Japan Clinical Research Support Unit (Tokyo, Japan). All participants remained anonymous, and the study was conducted according to Japanese privacy protection laws and ethical guidelines for epidemiological studies published by the Ministry of Education, Science, and Culture and the Ministry of Health, Labor, and Welfare. The study protocol was approved by the ethics committee in Fukushima Medical University (No. 1485).

The SHC has been previously described [6], [28]. Briefly, it is a new healthcare strategy initiated by the Japanese Government in 2008 for the early diagnosis and intervention of metabolic syndrome. The proportion of men to women in the SHC does not necessarily reflect the national population. This is because the SHC is designed for people who have National Health Insurance, or dependents (e.g., spouse) of salaried workers who have health insurance. In this system, participants answer a self-administered questionnaire that covers medical history, smoking habits, alcohol intake, exercise habits, and eating patterns. Trained staff then measure the height, weight, blood pressure, and waist circumference of each participant, after which serum and spot urine samples are collected. BMI is calculated by dividing body weight in kilograms by the square of height in meters. Blood samples are analyzed using an automated clinical chemical analyzer within 24 h of sampling. All blood analyses are conducted at a local, rather than a central, laboratory. Although the methods used for blood analyses are not calibrated between laboratories, analyses are performed according to the Japan Society of Clinical Chemistry-recommended methods for laboratory tests, which have been widely adopted by laboratories across Japan [29]. Participants diagnosed with metabolic syndrome are obligated to receive repeated lifestyle guidance over a six-month period after an annual health examination.

**Figure 1:**
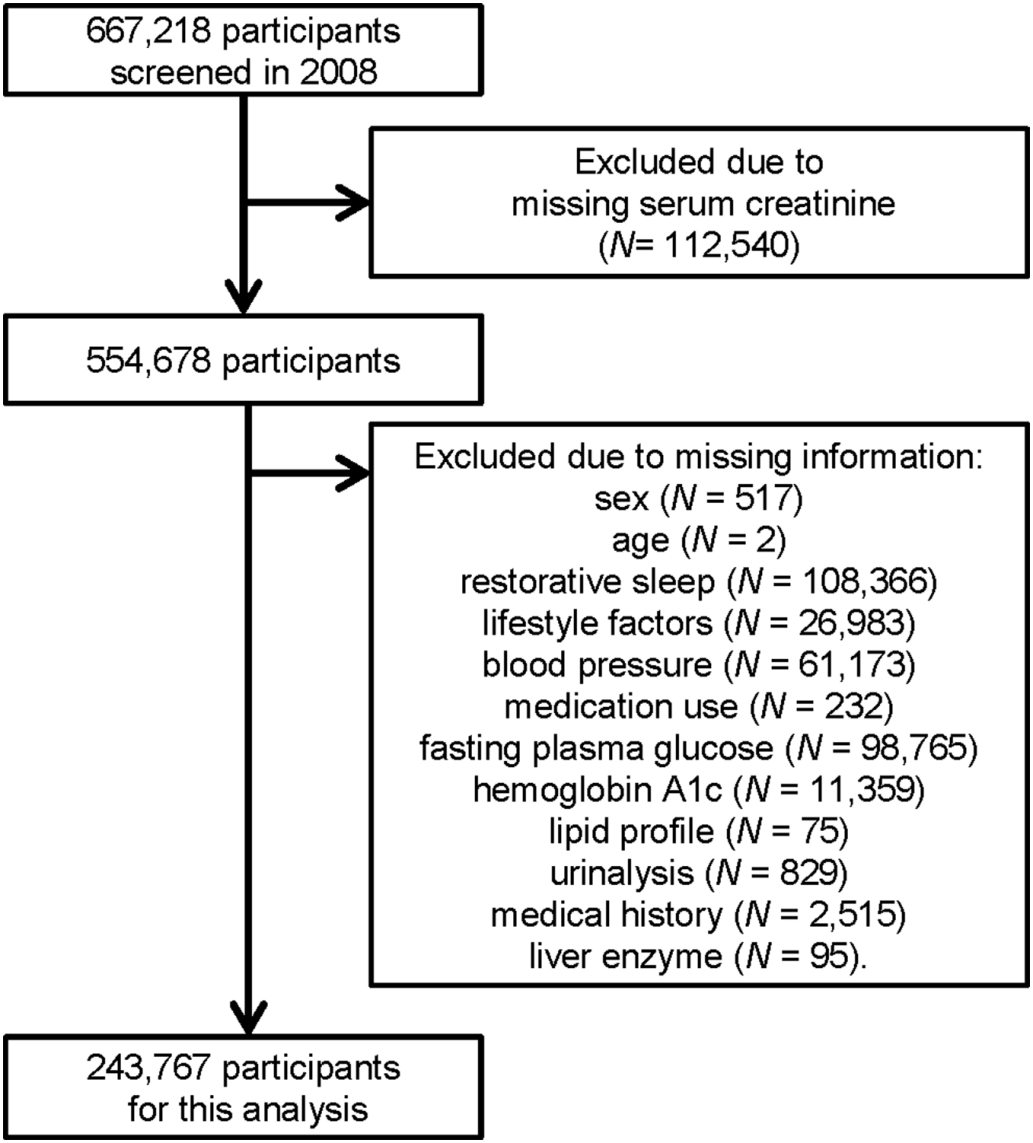
Flow chart of participant selection. Of the 667,218 SHC participants screened in 2008, we excluded anyone with missing information, resulting in a final sample size of 243,767.

Participants from 40 to 74 years of age without missing information were included in this study. The complete selection process is presented in Figure 1.

### Primary outcome

The primary outcome was NRS, which was assessed using this question from the self-administered questionnaire: ‘Do you feel refreshed after a night’s sleep?’ Participants answered either yes or no. NRS was considered present when the answer was ‘no’.

### Lifestyle factors and covariates

For each lifestyle factor (smoking, BMI, alcohol intake, exercise habits, and eating patterns), we created a binary low-risk variable in which participants were given a score of 1 if they met the criteria for low risk or a score of 0 if otherwise, based on previous research [6]. The a priori definition of low risk was based on current literature, recommended guidelines, and realistically obtainable levels within the general population. We calculated a healthy lifestyle score by adding the total number of lifestyle factors for which each participant was at low risk. The score ranged from 0 (least healthy) to 5 (most healthy).

For smoking, we defined low risk as currently not smoking. Optimal body weight was defined as a BMI of <25 kg/m^2^, the standard World Health Organization cutoff for healthy weight. For alcohol, average daily consumption over 20 g was considered high risk. Alcohol consumption was assessed by the following questions: “How often do you drink alcohol (sake, shochu [distilled spirits], beer, liquor, etc.)?” to which participants responded by selecting (1) every day, (2) sometimes, or (3) rarely (can’t drink); and “How much do you drink a day, in terms of glasses of refined sake? (A glass [180 mL] of refined sake is equivalent to a medium bottle [500 mL] of beer, 80 mL of shochu (alcohol content 35 percent), a glass [double, 60 mL] of whiskey, and 2 glasses [240 mL] of wine),” to which participants responded by selecting (1) <1 drink per day, (2) 1–2 drinks per day, (3) 2–3 drinks per day, or (4) ≥3 drinks per day’. The ethanol content per drink was calculated to be equivalent to 20 g. For exercise habits, two questions were asked: ‘Are you in the habit of exercising to sweat lightly for over 30 minutes each time, two times weekly, for over a year?’ and ‘In your daily life, do you walk or do any equivalent amount of physical activity for more than one hour a day?’ Low risk patients were defined as those who answered ‘yes’ to both questions on the basis of a current Japanese guideline [30]. For eating patterns, two questions were asked: ‘Do you skip breakfast more than three times a week?’ and ‘Do you eat snacks after supper more than three times a week?’ Low risk patients were defined as those who answered ‘no’ to both questions.

Hemoglobin A1c (HbA1c) was estimated as a National Glycohemoglobin Standardization Program equivalent value using the following equation [31]: HbA1c (%) = HbA1c (Japan Diabetes Society) (%)+0.4%. Diabetes was defined in accordance with American Diabetes Association guidelines [32] as a fasting plasma glucose concentration of 126 mg/dL or higher, HbA1c of 6.5% or higher, or self-reported use of anti-hyperglycemic drugs. Hypertension was defined as using antihypertensive medications, a systolic blood pressure ≥140 mmHg, a diastolic blood pressure ≥90 mmHg, or both. Hypercholesterolemia was defined as using cholesterol-lowering medications, a low-density lipoprotein (LDL) cholesterol level ≥140 mg/dL, or both. Chronic kidney disease was defined as proteinuria in urinalysis, a glomerular filtration rate (GFR) less than 60 mL/min/1.73 m^2^, or both [33]. Proteinuria was defined as a dipstick urinalysis score of 1+ or greater (equivalent to ≥30 mg/dL) because of poor discrimination between negative and trace positive dipstick readings [34]. Estimated GFR was calculated using the Japanese equation [35].

### Statistical analysis

Data were analyzed separately by sex. First, we calculated the prevalence of NRS stratified by age categories. Age was categorized as 40–49, 50–59, 60–69, and 70–74 years. Second, we analyzed clinical and laboratory parameters stratified by the presence or absence of NRS. The chi-square test, Student’s t-test, and Mann-Whitney U test were used to assess differences among participant characteristics in relation to NRS. Spearman and Pearson correlation coefficients were calculated to evaluate the relationship among each independent variable. To evaluate the association between prevalent NRS and each variable of the healthy lifestyle score, multivariable-adjusted odds ratios (ORs) and their corresponding 95% confidence intervals (CIs) were calculated using the category conventionally believed to be most healthy as the reference group. Data were initially adjusted for age. Next, we added age, hypertension, diabetes, hypercholesterolemia, and chronic kidney disease to multivariate models (Model 1). Finally, we added history of stroke, heart disease, and renal failure to Model 1 (Model 2).

To assess the robustness of the main results, we conducted several subsidiary analyses. First, subgroup analyses were stratified by age categories because age is associated with NRS. Healthy lifestyle scores from 0 (least healthy) to 1 (second-least healthy) were combined into one category because there were few cases. Age was also added as a continuous variable. Second, subgroup analyses were conducted among nonusers of medications to avoid variations in results due to medications. Nonusers of medications were defined as individuals who took no medications for diabetes, hypertension, and hypercholesterolemia. Finally, the analyses were repeated after excluding participants with obesity (BMI≥25 kg/m^2^) to enhance the association between healthy lifestyle and NRS, as obese participants could modify their lifestyle to lose weight. Because increased BMI may be a consequence rather than a component of an unhealthy lifestyle, another healthy lifestyle score was created to incorporate all variables except BMI. This score ranged from 0 to 4 points.

P<0.05 was considered statistically significant, and all tests were two-tailed. All analyses were performed with the SPSS for Windows statistical package (Version 18.0; SPSS, Chicago, IL, USA) and Stata/MP software (Version 12.1; Stata Corp, College Station, TX, USA).

## Results

### Participant flow

Of the 667,218 SHC participants screened in 2008, we excluded those who did not have serum creatinine levels measured (*n* = 112,540) because it is not mandatory for the SHC but is included independently in some areas. We also excluded anyone with missing information (*n* = 310,911), resulting in a final sample size of 243,767 (Figure 1). There were no substantial differences between included and excluded participants for characteristics such as prevalence of NRS, sex, age, and healthy lifestyle score (Table S1).

### Demographic characteristics of participants

Among 97,062 men and 146,705 women, 18,678 (19.2%) and 38,539 (26.3%) were identified as having NRS, respectively. Tables 1 and 2 present the associations between various clinical characteristics and NRS. Both male and female participants with NRS were younger, had higher prevalence of current smokers, higher BMI, lower prevalence of exercise habits, and higher prevalence of less healthy eating patterns. They were also more likely to have a history of heart or renal disease and less likely to have hypertension, diabetes, and chronic kidney disease. Some differences were observed between sexes. Women with NRS were less likely to have an adequate intake of daily alcohol, while this was more likely in men with NRS compared to those without NRS. In addition, the proportion of those with a history of stroke was significantly higher in women with NRS, but not in men with NRS.

**Table 1:** Clinical characteristics of male participants by restorative sleep achievement.

Characteristics	Total (*N* = 97,062)	Restorative sleep(*N* = 78,384 [80.8%])	Non-restorative sleep(*N* = 18,678 [19.2%])	*P* value
Age, years	63.9 (8.5)	64.4 (8.2)	61.7 (9.5)	<0.0001
Healthy lifestyle score, *n* (%)				<0.0001
0	484 (0.5)	360 (0.5)	124 (0.7)	
1	5,049 (5.2)	3,715 (4.7)	1,334 (7.1)	
2	16,986 (17.5)	13,140 (16.8)	3,846 (20.6)	
3	30,331 (31.3)	24,326 (31.0)	6,005 (32.2)	
4	31,355 (32.3)	25,735 (32.8)	5,620 (30.1)	
5	12,857 (13.3)	11,108 (14.2)	1,749 (9.4)	
Components of the healthy lifestyle score				
Current smoker, *n* (%)	25,359 (26.1)	20,214 (25.8)	5,145 (27.5)	<0.0001
Body mass index, kg/m^2^	23.6 (3.0)	23.6 (2.9)	23.7 (3.2)	0.05
Alcohol<20 g/day, *n* (%)	67,556 (69.6)	53,924 (68.8)	13,632 (73.0)	<0.0001
Regular exercise				
Exercise to sweat lightly, *n* (%)	46,069 (47.5)	39,055 (49.8)	7,014 (37.6)	<0.0001
Walking>1 hour/day, *n* (%)	53,950 (55.6)	45,253 (57.7)	8,697 (46.6)	<0.0001
Eating pattern				
Snacks after supper, *n* (%)	12,000 (12.4)	8,775 (11.2)	3,225 (17.3)	<0.0001
Skipping breakfast, *n* (%)	11,060 (11.4)	7,818 (10.0)	3,242 (17.4)	<0.0001
Past history, *n* (%)				
Stroke	4.938 (5.1)	4,003 (5.1)	935 (5.0)	0.59
Heart disease	7,964 (8.2)	6,285 (8.0)	1,679 (9.0)	<0.0001
Renal disease	552 (0.6)	417 (0.5)	135 (0.7)	0.002
Comorbidities, *n* (%)				
Hypertension	49,135 (50.6)	40,406 (51.5)	8,729 (46.7)	<0.0001
Diabetes	14,596 (15.0)	11,965 (15.3)	2,631 (14.1)	<0.0001
Hypercholesterolemia	33, 258 (34.3)	26,878 (34.3)	6,380 (34.2)	0.74
Chronic kidney disease	22,570 (23.3)	18,481 (23.6)	4,089 (21.9)	<0.0001
Medication, *n* (%)				
Antihypertensive drugs	30,756 (31.7)	25,382 (32.4)	5,374 (28.8)	<0.0001
Antidiabetic medication	6,649 (6.9)	5,457 (7.0)	1,192 (6.4)	0.005
Cholesterol-lowering drugs	10,779 (11.1)	8,856 (11.3)	1,923 (10.3)	<0.0001
Systolic pressure, mmHg	131 (17)	132 (17)	130 (17)	<0.0001
Diastolic pressure, mmHg	78 (11)	78 (11)	78 (11)	0.001
Fasting plasma glucose, mg per 100 mL	102 (25)	102 (24)	102 (26)	0.66
Hemoglobin A_1c_, %	5.79 (0.79)	5.79 (0.77)	5.78 (0.85)	0.02
LDL cholesterol, mg per 100 mL	120.9 (30.0)	120.8 (29.9)	121.1 (30.4)	0.21
Triglycerides, mg per 100 mL	107 (77, 154)	107 (77, 153)	107 (76, 155)	0.58
HDL cholesterol, mg per 100 mL	57 (15)	57 (15)	57 (15)	0.96
Creatinine, mg per 100 mL	0.85 (0.23)	0.85 (0.23)	0.84 (0.23)	0.03
eGFR, mL min^–1^ per 1.73 m^2^	74.4 (16.4)	74.2 (16.3)	75.6 (16.9)	<0.0001
Proteinuria, *n* (%)	7,670 (7.9)	6,157 (7.9)	1,513 (8.1)	0.26

Numbers in the table are means (standard deviation) for continuous variables except triglycerides (median and interquartile range) or numbers (percentages) for categorical variables.

LDL, low-density lipoprotein; HDL, high-density lipoprotein; eGFR, estimated glomerular filtration rate.

**Table 2:** Clinical characteristics of female participants by restorative sleep achievement.

Characteristics	Total(*N* = 146,705)	Restorative sleep(*N* = 108,166 [73.7%])	Non-restorativeSleep(*N* = 38,539 [26.3%])	*P* value
Age, years	63.7 (7.9)	64.2 (7.7)	62.6 (8.5)	<0.0001
Healthy lifestyle score, *n* (%)				<0.0001
0	63 (0.0)	35 (0.0)	28 (0.1)	
1	1,283 (0.9)	765 (0.7)	518 (1.3)	
2	9,909 (6.8)	6,502 (6.0)	3,407 (8.8)	
3	35,999 (24.5)	25,208 (23.3)	10,791 (28.0)	
4	71,637 (48.8)	53,100 (49.1)	18,537 (48.1)	
5	27,814 (19.0)	22,556 (20.9)	5,258 (13.6)	
Components of the healthy lifestyle score				
Current smoker, *n* (%)	9,763 (6.7)	6,655 (6.2)	3,108 (8.1)	<0.0001
Body mass index, kg/m^2^	22.7 (3.4)	22.7 (3.3)	22.6 (3.5)	<0.0001
Alcohol<20 g/day, *n* (%)	142,216 (96.9)	105,038 (97.1)	37,178 (96.5)	<0.0001
Regular exercise				
Exercise to sweat lightly, *n* (%)	58,932 (40.2)	46,404 (42.9)	12,528 (32.5)	<0.0001
Walking>1 hour/day, *n* (%)	76,043 (51.8)	58,492 (54.1)	17,551 (45.5)	<0.0001
Eating pattern				
Snacks after supper, *n* (%)	20,361 (13.9)	13,630 (12.6)	6,731 (17.5)	<0.0001
Skipping breakfast, *n* (%)	11,791 (8.0)	7,429 (6.9)	4,362 (11.3)	<0.0001
Past history, *n* (%)				
Stroke	3.902 (2.7)	2,813 (2.6)	1,089 (2.8)	0.02
Heart disease	7,606 (5.2)	5,271 (4.9)	2,335 (6.1)	<0.0001
Renal disease	632 (0.4)	436 (0.4)	196 (0.5)	0.007
Comorbidities, *n* (%)				
Hypertension	62,034 (42.3)	46,822 (43.3)	15,212 (39.5)	<0.0001
Diabetes	11,623 (7.9)	8,625 (8.0)	2,998 (7.8)	<0.0001
Hypercholesterolemia	74,371 (50.7)	55,682 (51.5)	18,689 (48.5)	<0.0001
Chronic kidney disease	21,762 (14.8)	16,146 (14.9)	5,616 (14.6)	<0.0001
Medication, *n* (%)				
Antihypertensive drugs	39,592 (27.0)	29,884 (27.6)	9,708 (25.2)	<0.0001
Antidiabetic medication	5,373 (3.7)	3,960 (3.7)	1,413 (3.7)	0.96
Cholesterol-lowering drugs	28,802 (19.6)	21,771 (20.1)	7,031 (18.2)	<0.0001
Systolic pressure, mmHg	128 (18)	129 (18)	130 (18)	<0.0001
Diastolic pressure, mmHg	75 (11)	75 (11)	75 (11)	<0.0001
Fasting plasma glucose, mg per 100 mL	95 (18)	95 (17)	95 (19)	0.03
Hemoglobin A_1c_, %	5.71 (0.59)	5.72 (0.58)	5.70 (0.60)	<0.0001
LDL cholesterol, mg per 100 mL	130.0 (30.3)	130.2 (30.2)	129.2 (30.8)	<0.0001
Triglycerides, mg per 100 mL	92 (68, 127)	92 (69, 127)	91 (67, 126)	<0.0001
HDL cholesterol, mg per 100 mL	65.7 (16.0)	65.5 (16.0)	66.3 (16.2)	<0.0001
Creatinine, mg per 100 mL	0.63 (0.15)	0.63 (0.15)	0.63 (0.16)	0.005
eGFR, mL min^–1^ per 1.73 m^2^	75.6 (15.9)	75.4 (15.9)	76.3 (16.1)	<0.0001
Proteinuria, *n* (%)	5,777 (3.9)	4,169 (3.9)	1,608 (4.2)	0.006

Numbers in the table are means (standard deviation) for continuous variables except triglycerides (median and interquartile range) or numbers (percentages) for categorical variables.

LDL, low-density lipoprotein; HDL, high-density lipoprotein; eGFR, estimated glomerular filtration rate.

### Associations between the healthy lifestyle score and NRS

**Figure 2:**
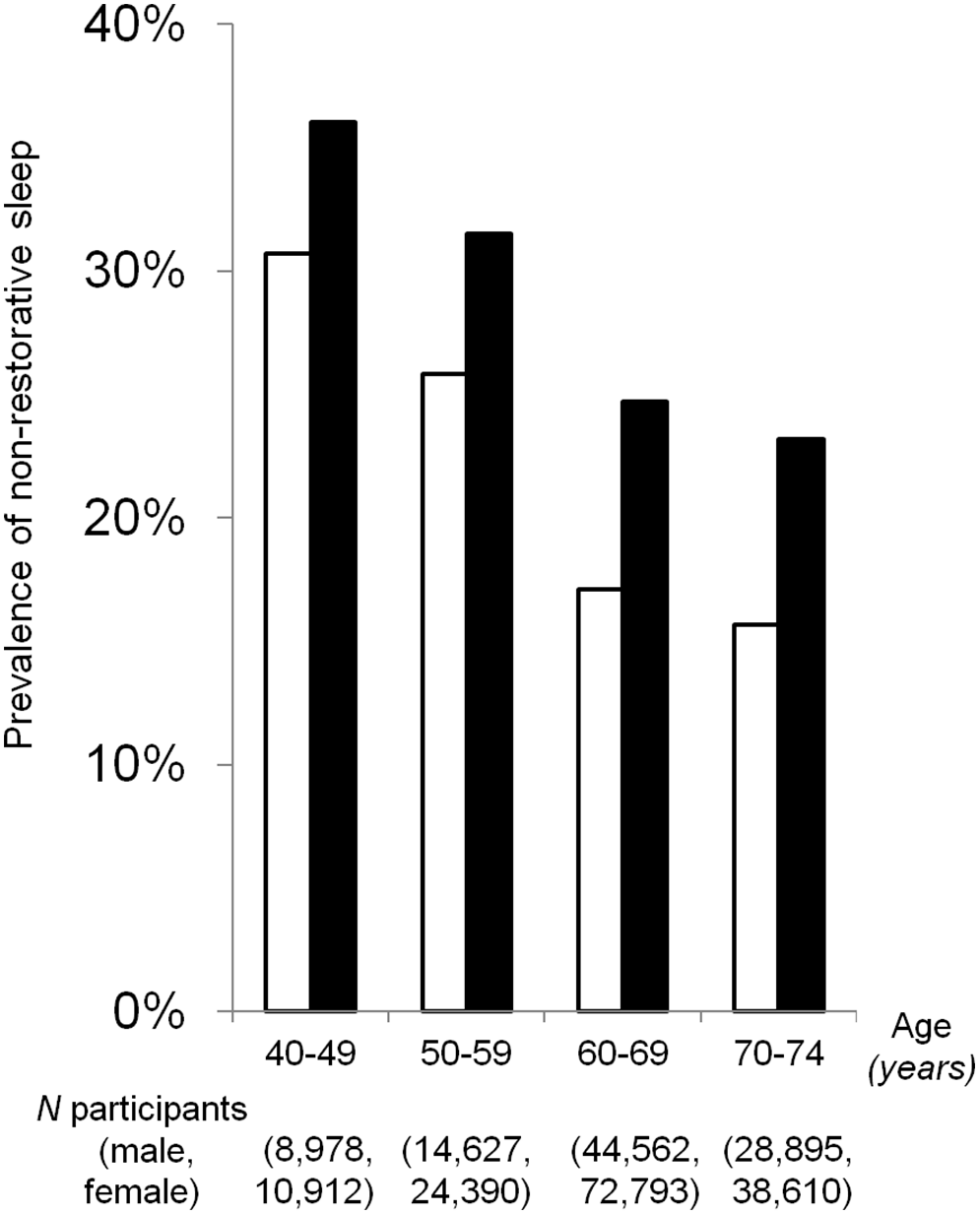
Prevalence of non-restorative sleep by sex and age. Trends were significant for both males (□; *P*<0.0001) and females (▪; *P*<0.0001).

**Figure 3:**
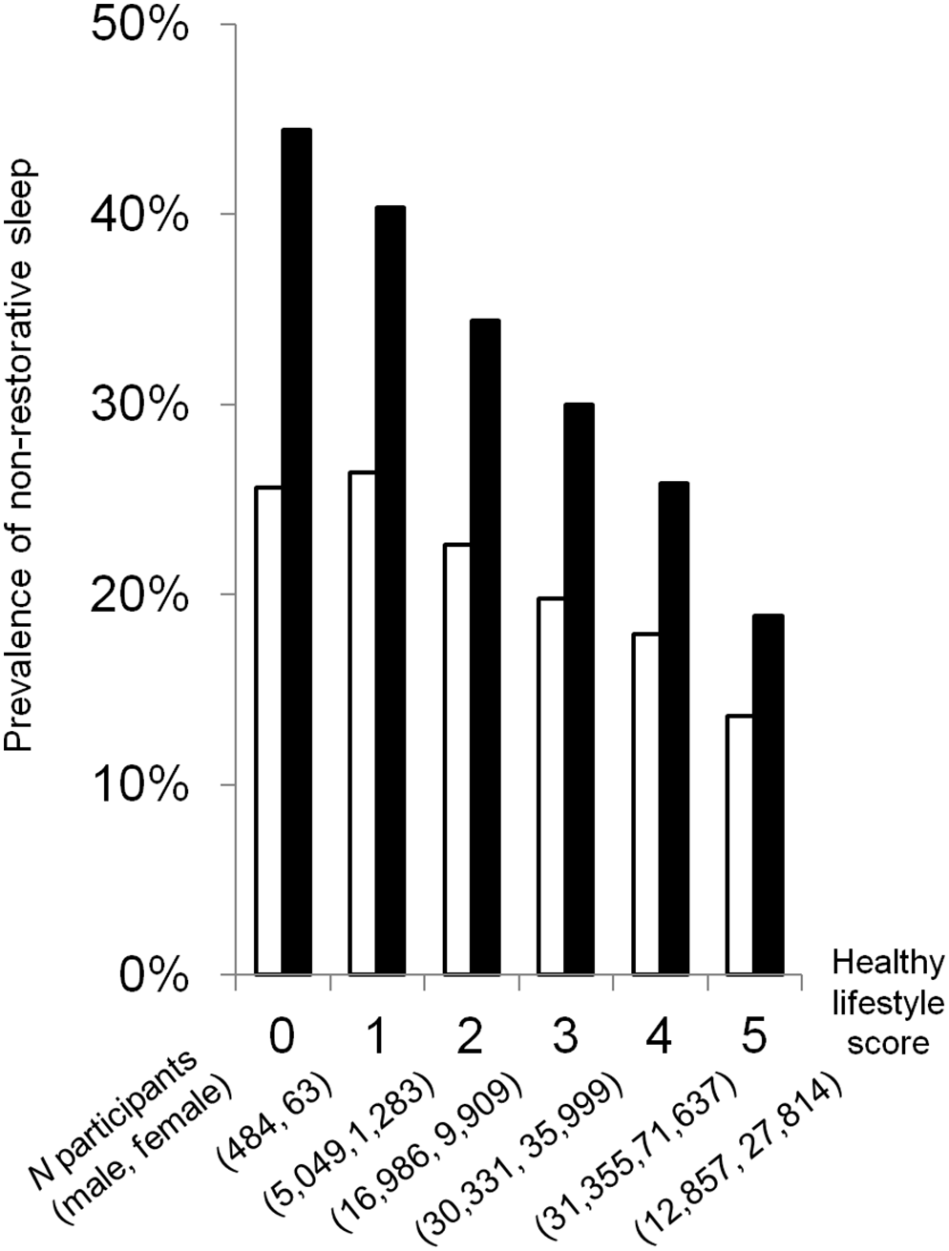
Prevalence of non-restorative sleep by healthy lifestyle score. Trends were significant for both males (□; *P*<0.0001) and females (▪; *P*<0.0001).

The prevalence of NRS decreased with increasing age for both men and women (*P* for trend <0.0001, Figure 2), and was lower among men than women for all age groups. An inverse, dose-response relationship was observed between healthy lifestyle scores and prevalence of NRS for both male and female participants (*P* for trend <0.0001, Figure 3).

When each variable of the healthy lifestyle score was considered individually, less healthy eating patterns and no regular exercise were associated with a higher prevalence of NRS (Table 3), but there were no apparent associations between BMI and the prevalence of NRS. Some differences were observed between men and women. Current smokers were associated with a higher prevalence of NRS in women and a lower prevalence in men. In addition, alcohol consumption was not associated with NRS in women but was associated with a lower prevalence in men.

**Table 3:** Multivariate analysis of the relationship between categories from the healthy lifestyle score and prevalence of non-restorative sleep (*N* = 243,767).

Variable	Male (*N* = 97,062)		Female (*N* = 146,705)	
	Age-adjustedodds ratio(95%CI)	Multivariateodds ratio^a^(95%CI)	Age-adjustedodds ratio(95%CI)	Multivariateodds ratio^a^(95%CI)
Categories				
Current smoker				
No (ref)	1.00	1.00	1.00	1.00
Yes	0.90 (0.87–0.93)****	0.90 (0.87–0.93)****	1.09 (1.04–1.14)****	1.09 (1.04–1.14)****
Body mass index				
<25 m/kg^2^ (ref)	1.00	1.00	1.00	1.00
≥25 m/kg^2^	0.97 (0.93–1.00)	0.97 (0.94–1.01)	0.98 (0.95–1.00)	0.99 (0.96–1.02)
Alcohol consumption				
<20 g/day (ref)	1.00	1.00	1.00	1.00
≥20 g/day	0.83 (0.80–0.86)****	0.83 (0.80–0.86)****	1.03 (0.96–1.10)	1.03 (0.96–1.10)
Regular exercise				
Yes (ref)	1.00	1.00	1.00	1.00
No	1.57 (1.51–1.62)****	1.57 (1.51–1.63)****	1.52 (1.47–1.58)****	1.52 (1.48–1.56)****
Eating pattern				
Healthy (ref)	1.00	1.00	1.00	1.00
Less healthy	1.54 (1.48–1.60)****	1.54 (1.48–1.60)****	1.44 (1.40–1.48)****	1.44 (1.40–1.48)****
Age				
40–49 years	1.96 (1.85–2.08)****	1.95 (1.84–2.07)****	1.56 (1.48–1.63)****	1.50 (1.43–1.57)****
50–59 years	1.63 (1.55–1.71)****	1.62 (1.54–1.71)****	1.35 (1.30–1.40)****	1.32 (1.27–1.37)****
60–69 years	1.09 (1.04–1.13)****	1.09 (1.04–1.13)****	1.06 (1.03–1.09)****	1.05 (1.02–1.09)**
70–74 years (ref)	1.00	1.00	1.00	1.00
Hypertension		0.96 (0.93–1.00)*		0.94 (0.91–0.96)****
Diabetes mellitus		0.99 (0.95–1.04)		1.06 (1.02–1.11)**
Hypercholesterolemia		0.97 (0.93–1.00)		0.95 (0.92–0.97)****
Chronic kidney disease		1.04 (1.00–1.08)		1.04 (1.00–1.08)*

a Adjusted for age (years), sex, hypertension, diabetes, hypercholesterolemia, and chronic kidney disease.

Definitions of these factors are described in the text.

*P<0.05,

**P<0.01,

***P<0.001,

****P<0.0001.

Tables 4 and 5 show that participants with a score of 0 (least healthy) had an age-adjusted OR of 1.56 (95% CI, 1.26–1.93) for men and 2.76 (95% CI, 1.68–4.56) for women, compared to those with a score of 5 (most healthy). Additional adjustments for potential consequences of an unhealthy lifestyle (i.e., hypertension, diabetes mellitus, hypercholesterolemia, and chronic kidney disease) only partially changed risk (OR for men: 1.59, 95% CI, 1.29–1.97; OR for women: 2.88, 95% CI, 1.74–4.76). This association was not changed by additional adjustments for history of stroke, heart disease, and renal failure (Model 2).

**Table 4:** Odds ratios for the association between the healthy lifestyle score and prevalent non-restorative sleep in men (*N* = 97,062).

Healthy lifestyle score	Unadjusted	Age-adjusted	Model 1	Model 2
0	2.19 (1.77–2.70)****	1.56 (1.26–1.93)****	1.59 (1.29–1.97)****	1.60 (1.29–1.98)****
1	2.28 (2.10–2.47)****	1.73 (1.59–1.88)****	1.76 (1.62–1.91)****	1.77 (1.63–1.92)****
2	1.86 (1.75–1.98)****	1.54 (1.44–1.64)****	1.56 (1.46–1.66)****	1.56 (1.46–1.66)****
3	1.57 (1.48–1.66)****	1.40 (1.32–1.48)****	1.41 (1.33–1.49)****	1.41 (1.33–1.50)****
4	1.39 (1.31–1.47)****	1.31 (1.24–1.39)****	1.32 (1.24–1.40)****	1.32 (1.24–1.40)****
5 (ref)	1.00	1.00	1.00	1.00

Model 1: adjusted for age (years), hypertension, diabetes, hypercholesterolemia, and chronic kidney disease.

Model 2: adjusted Model 1 plus history of stroke, heart disease, and renal failure.

*P<0.05,

**P<0.01,

***P<0.001,

****P<0.0001.

**Table 5:** Odds ratios for the association between the healthy lifestyle score and prevalent non-restorative sleep in women (*N* = 146,705).

Healthy lifestyle score	Unadjusted	Age-adjusted	Model 1	Model 2
0	3.43 (2.09–5.65)****	2.76 (1.68–4.56)****	2.88 (1.74–4.76)****	2.88 (1.74–4.75)****
1	2.91 (2.59–2.26)****	2.43 (2.16–2.73)****	2.48 (2.21–2.79)****	2.47 (2.20–2.78)****
2	2.25 (2.14–2.37)****	2.00 (1.90–2.11)****	2.04 (1.94–2.15)****	2.03 (1.93–2.14)****
3	1.84 (1.77–1.91)****	1.71 (1.65–1.78)****	1.74 (1.67–1.81)****	1.73 (1.67–1.80)****
4	1.50 (1.45–1.55)****	1.44 (1.39–1.49)****	1.45 (1.40–1.50)****	1.44 (1.39–1.49)****
5 (ref)	1.00	1.00	1.00	1.00

Model 1: adjusted for age (years), hypertension, diabetes, hypercholesterolemia, and chronic kidney disease.

Model 2: adjusted Model 1 plus history of stroke, heart disease, and renal failure.

*P<0.05,

**P<0.01,

***P<0.001,

****P<0.0001.

**Figure 4:**
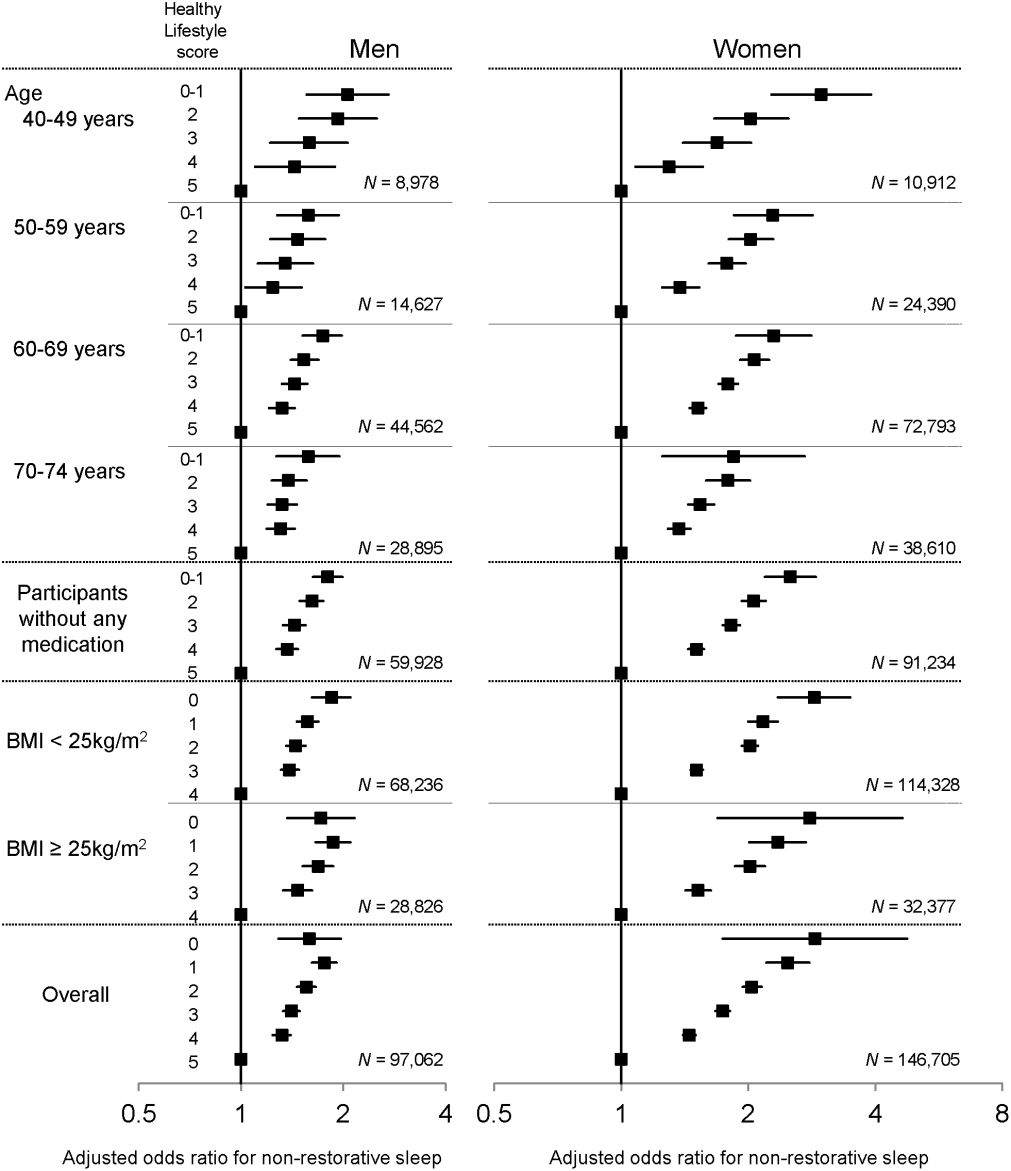
Subgroup analysis. Forest plot shows odds ratio with 95% confidence interval for the association between a healthy lifestyle score and prevalent non-restorative sleep in subgroups and in the entire study population. All analyses were adjusted for the following covariates: age (in years), hypertension, diabetes, hypercholesterolemia, and chronic kidney disease.

When stratified by age categories, the association between a healthy lifestyle score and prevalence of NRS was similar when compared with the entire study population for both men and women (Figure 4). Among participants who currently took no medications for diabetes, hypertension, or hypercholesterolemia, a similar association was also observed for both sexes. Furthermore, the associations were consistent among obese and non-obese participants.

## Discussion

Our findings support the hypothesis that a combination of unhealthy lifestyle factors is associated with inadequate sleep. A combination of healthy lifestyle factors was associated with a decreased prevalence of NRS in both men and women of any age, even after adjusting for comorbidities such as hypertension, diabetes mellitus, hypercholesterolemia, chronic kidney disease, and history of stroke, heart disease, and renal failure. Although further study is needed to strengthen the association between the combined lifestyle factors and inadequate sleep, these findings raise the possibility that a healthy lifestyle could not only reduce the risk of developing several diseases but also improve sleep quality. If healthy lifestyle factors can provide restorative sleep, gaining satisfying rest would be a strong motivator for lifestyle modification.

Insomnia is an important public health issue [36] characterized by nighttime sleep problems. These may be manifest as difficulties in initiating or maintaining sleep, NRS, a combination of these complaints, or daytime symptoms [37]. Although there is a lack of consistency in the definition of NRS, and reports on NRS have been less extensive compared with other symptoms of primary insomnia (i.e., sleep latency and total sleep time), mounting evidence suggests that NRS is a frequent symptom observed in the general population. NRS prevalence was reported to be 10.8% in the non-institutionalized general population in seven European countries (France, the United Kingdom, Germany, Italy, Portugal, Spain, and Finland) [38], 35% in the participants of the Atherosclerosis Risk in Community Study in the United States [39], and 14.8% in the general South Korean population [40]. Although our methodology and questionnaire were not the same as those used in these studies, our findings were consistent in that NRS is frequently observed in the general Japanese population. The prevalence of NRS was higher in women than in men and decreased with age in our study. These results are in line with previous studies from Europe [38], the United States [36], [39], South Korea [40], as well as an international survey conducted in Finland, Greece, Jordan and Lebanon, Morocco, Mexico, the Philippines, Portugal, Sweden, and Switzerland [41]. Although the prevalence of insomnia differs from one country to another [40], a decrease in NRS prevalence with age is commonly observed. This suggests that some common risk factors may be shared among young people with different racial, ethnic, cultural, and environmental background.

With regard to individual lifestyle factors, we found associations between NRS and physical inactivity or unhealthy eating patterns. Both physical activity and a healthy diet are known to be associated with sleep quality. A high level of exercise is related to better sleep patterns such as higher sleep quality, shortened sleep latency, and fewer awakenings during the night [42], while lack of habitual exercise is associated with more reported sleep complaints [43]–[44]. Meanwhile, skipping breakfast and a regular habit of snacking are more common in individuals with short sleep duration than in those with normal sleep duration [45]. A randomized crossover study showed that skipping breakfast in a nocturnal lifestyle, i.e., sleeping at 1∶30 a.m. and waking at 8∶30 a.m., was associated with decreased secretion of melatonin and leptin [46], suggesting that those lifestyle factors might be both a cause and a consequence of inadequate sleep. Although information is limited about NRS and physical inactivity or eating patterns, our findings are in line with these studies.

We found no association between NRS and BMI. Although obesity is associated with sleep apnea, little is known about its connection to NRS. Previous literature has shown that BMI is not associated with the prevalence of NRS, except in underweight individuals (BMI<20 kg/m^2^) who had a higher prevalence of NRS than those with normal BMI (20–24 kg/m^2^) [40]. A possible reason for this discrepancy is that use of a binary variable in our analysis did not detect an association between being underweight and having NRS that might only be observed when analyzed quantitatively.

Notably, sex differences were observed for smoking and alcohol consumption. Although smoking was associated with increases in NRS in women, the opposite relationship was observed in men, and adequate alcohol consumption was associated with increases in NRS among only male participants. It has generally been found that both alcohol consumption and smoking are associated with increases in sleep disorders [47]–[49], but associations between NRS and alcohol consumption or smoking are still controversial. Smoking has been associated with NRS in some studies [40], [48] but not in another [39]. While alcohol consumption is not associated with NRS [39], some studies have found that alcohol abuse [50] and alcohol dependence [40] are connected to NRS. These inconsistencies may be primarily due to the different definitions of NRS, which only represents a single dimension or item of sleep symptoms [36]. It is also possible that using the binary variable in our study influenced results. Although associations between NRS and alcohol or smoking differed between men and women, the combined effect of healthy lifestyle factors on NRS were similar for both, suggesting that lifestyle factors cooperate with one another and are important for restorative sleep. Furthermore, a comprehensive analysis of healthy lifestyle may capture influence of individual factors better than analyses based on a single factor, given the complexity and multiple dimensions of habitual health behaviors. Further investigation is needed to determine whether critical thresholds exist for each lifestyle factor and to establish combined effects and interaction effects as well as individual effects.

Patients with NRS were recently reported to have higher C-reactive protein (CRP) levels, a marker for systemic inflammation, than those without NRS [51]. Elevated CRP has also been associated with lifestyle risk factors such as obesity [52], physical inactivity [53], cigarette smoking [54], and alcohol consumption [55], [56]. These findings together suggest that participants with NRS have higher CRP levels due to unhealthy lifestyle behaviors, although no information about CRP was available in our study. It is possible that sleep deprivation [57], [58] or stress [59] leads to increased CRP levels. Further investigation is warranted to clarify these aspects of the relationship between NRS and lifestyle factors.

Our study has several limitations. First, a selection bias of subjects might exist. Because participants in this cohort received annual physical checkups, they might be more health-conscious than the average Japanese population. Second, NRS was determined solely based on self-reported information and may not be accurate. A single retrospective item has limitations that need to be addressed, such as recall bias and demand characteristics. In addition, frequency (i.e., more than three times per week) and sleep duration were not included in the questionnaire used to assess NRS. However, there is no reliable and well-validated patient-reported outcome instrument currently available for evaluating NRS [27]. Third, we cannot exclude the possibility that residual confounding factors exist, which were not measured in the present study, such as marital status [38], educational level [36], [50], employment status [38], work schedule [50], level of stress [50], psychiatric disorders [38], and use of sleep medications. Whether these factors affect the relationship between lifestyle factors and NRS should be assessed in the future. Fourth, we gave equal weight to each lifestyle factor to achieve the main purpose of the study. That may have resulted in conservative estimates for multiple lifestyle factors. Fifth, the nutritive content in diet could not be evaluated due to lack of information. Evidence regarding the associations between diet and NRS is scarce, although a cross-sectional study using data from the National Health and Nutrition Examination Survey (NHANES) from the United States has shown that NRS is positively associated with butanoic acid, moisture, cholesterol, and negatively associated with calcium, vitamin C, and water [60]. Sixth, odds ratios do not approximate well to the relative risk when the effect sizes are large and the prevalence of the outcome of interest is high [61]. In our study, the prevalence of NRS was relatively high, whereas the effect sizes were not large. In addition, qualitative judgments based on interpreting odds ratios as though they were relative risks are unlikely to be seriously in error [61]. Therefore, we consider that our results demonstrate important effects. Finally, the cross-sectional study design limited our ability to determine the direction of the association or causality. NRS might be the cause, rather than the consequence, of one or more unhealthy lifestyle factors.

Our study also has several strengths. First, it was a large-scale cross-sectional study with participants from all over Japan. Second, this is the first report, to the best of our knowledge, to demonstrate the prevalence of NRS in the general Japanese population and assess the association between combined lifestyle factors and NRS.

In conclusion, a combination of healthy lifestyle factors was associated with an increase in restorative sleep independently of age, sex, and comorbidities in the general Japanese population. Further studies are needed to establish whether general lifestyle modification improves restorative sleep.

## Supporting Information

Table S1 Clinical characteristics of participants included and excluded in the analysis. Numbers in the table are means (standard deviation) for continuous variables except triglycerides (median and interquartile range) or numbers (percentages) for categorical variables. Note that the percentages are computed based only on the total number of non-missing cases. LDL, low-density lipoprotein; HDL, high-density lipoprotein; eGFR, estimated glomerular filtration rate.
(DOC)
