# Supplementary material for: Association between Combined Lifestyle Factors and Non-Restorative Sleep in Japan: A Cross-Sectional Study Based on a Japanese Health Database
Source: PLoS One. 2014 Sep 30;9(9):e108718. doi: 10.1371/journal.pone.0108718 (PMC4182544; doi:10.1371/journal.pone.0108718)
Supplement: Table S1 — Clinical characteristics of participants included and excluded in the analysis. Numbers in the table are means (standard deviation) for continuous variables except triglycerides (median and interquartile range) or numbers (percentages) for categorical variables. Note that the percentages are computed based only on the total number of non-missing cases. LDL, low-density lipoprotein; HDL, high-density lipoprotein; eGFR, estimated glomerular filtration rate. (DOC) [file pone.0108718.s001.doc]

Table S1. Clinical characteristics of participants included and excluded in the analysis.

| Parameter | Included (*N* = 243,767) | Excluded (*N*= 423,451) |
| --- | --- | --- |
| Non-restorative sleep, *n* (%) | 57,217 (23.5) | 66,316 (22.7) |
| Male sex, *n* (%) | 97,062 (39.8) | 173,532 (41.0) |
| Age, years | 63.8 (8.2) | 63.5 (8.3) |
| Healthy lifestyle score, *n* (%) |  |  |
| 0 | 547 (0.2) | 608 (0.2) |
| 1 | 6,332 (2.6) | 6,832 (2.5) |
| 2 | 26,895 (11.0) | 30,322 (11.3) |
| 3 | 66,330 (27.2) | 75,905 (28.3) |
| 4 | 102,992 (42.3) | 114,501 (42.6) |
| 5 | 40,671 (16.7) | 40,301 (15.0) |
| Components of the healthy lifestyle score |  |  |
| Current smoker, *n* (%) | 35,122 (14.4) | 60,874 (15.3) |
| Body mass index, kg/m^2^ | 23.9 (3.1) | 23.3 (3.0) |
| Alcohol < 20 g/day, *n* (%) | 209,772 (86.1) | 294,780 (86.0) |
| Regular exercise |  |  |
| Exercise to sweat lightly, *n* (%) | 105,001 (43.1) | 117,649 (40.0) |
| Walking > 1 hour/day, *n* (%) | 129,993 (53.3) | 152,863 (52.5) |
| Eating pattern |  |  |
| Snacks after supper, *n* (%) | 32,361 (13.3) | 38,181 (13.1) |
| Skipping breakfast, *n* (%) | 22,851 (9.4) | 24,014 (8.3) |
| Past history, *n* (%) |  |  |
| Stroke | 8,840 (3.6) | 15,097 (4.4) |
| Heart disease | 15,570 (6.4) | 22,534 (6.7) |
| Renal disease | 1,184 (0.5) | 5,040 (1.5) |
| Comorbidities, *n* (%) |  |  |
| Hypertension | 111,169 (45.6) | 161,274 (47.8) |
| Diabetes | 26,219 (10.8) | 15,362 (11.2) |
| Hypercholesterolemia | 107,629 (44.2) | 170,807 (42.3) |
| Chronic kidney disease | 44,332 (18.2) | 54,126 (17.6) |
| Medication, *n* (%) |  |  |
| Antihypertensive drugs | 70,348 (28.9) | 125,229 (31.0) |
| Antidiabetic medication | 12,022 (4.9) | 22,432 (5.6) |
| Cholesterol-lowering drugs | 39,581 (16.2) | 64,859 (16.0) |
| Systolic BP, mmHg | 132 (18) | 130 (18) |
| Diastolic BP, mmHg | 77 (11) | 76 (11) |
| Fasting plasma glucose, mg per 100 mL | 98 (21) | 98 (20) |
| Hemoglobin A_1c_, % | 5.7 (0.7) | 5.7 (0.7) |
| LDL cholesterol, mg per 100 mL | 126 (31) | 125 (31) |
| HDL cholesterol, mg per 100 mL | 62 (16) | 62 (16) |
| Triglycerides, mg per 100 mL | 97 (71, 137) | 103 (74, 148) |
| Creatinine, mg per 100 mL | 0.72 (0.22) | 0.72 (0.26) |
| eGFR, mL min^–1^ per 1.73 m^2^ | 75.2 (16.1) | 75.8 (16.8) |
| Proteinuria, *n* (%) | 13,447 (5.5) | 19,862 (4.8) |
